# Optimizing Epoxy Nanocomposites with Oxidized Graphene Quantum Dots for Superior Mechanical Performance: A Molecular Dynamics Approach

**DOI:** 10.1021/acsomega.5c00013

**Published:** 2025-04-04

**Authors:** Prathamesh P. Deshpande, Robert Chan-Jobe, Josh Kemppainen, Gregory M. Odegard, Ozgur Keles

**Affiliations:** † Department of Chemical and Materials Engineering, San Jose State University, San Jose, California 95192, United States; ‡ Department of Mechanical Engineering-Engineering Mechanics, Michigan Technological University, Houghton, Michigan 49931, United States; § Department of Mechanical Engineering and Engineering Science, University of North Carolina at Charlotte, Charlotte, North Carolina 28223, United States

## Abstract

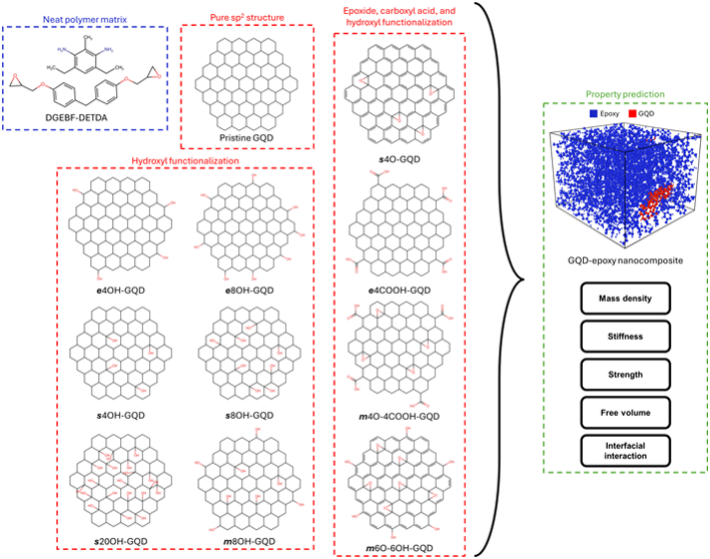

Due to their excellent mechanical properties, epoxy composites are widely used in low-density applications. However, the brittle epoxy matrix often serves as the principal failure point. Matrix enhancements can be achieved by optimizing polymer combinations to maximize intermolecular interactions or by introducing fillers. While nanofillers such as clay, rubber, carbon nanotubes, and nanoplatelets enhance mechanical properties, they can lead to issues like agglomeration, voids, and poor load transfer. Quantum dots, being the smallest nanofillers, offer higher dispersion and the potential to promote intermolecular interactions, enhancing stiffness, strength, and toughness simultaneously. This study employed molecular dynamics simulations to design graphene quantum dot (GQD) reinforced epoxy nanocomposites. By functionalizing GQDs with oxygen-based groups—hydroxyl, epoxide, carboxyl, and mixed chemistries—their effects on the mechanical properties of nanocomposites were systematically evaluated. Results show that hydroxyl-functionalized GQDs provide optimal performance, increasing stiffness and yield strength by 18.4 and 56.1%, respectively. Structural analysis reveals that these GQDs promote a closely packed molecular configuration, resulting in reduced free volume.

## 1 Introduction

Epoxy-based composites are used in a plethora of applications, including adhesives, piping, electronics, and structures. These composites are popular due to their superior material properties, such as high specific strength and stiffness. The epoxy matrix, a thermosetting polymer, forms the main component of the composite and is responsible for transferring load within the material. However, the cross-linked network of epoxy polymers often makes the neat matrix the focal point of failure due to poor crack resistance. Therefore, superior polymer matrix composites require thermoset matrices with better mechanical properties. Various studies have explored different approaches to enhance the toughness of thermosets. These approaches include adding second phase polymers (e.g., rubber, thermoplastics, core–shell polymers, liquid crystals, hyperbranched polymers) and other second phases, such as fibers, nanoplatelets, nanotubes, and quantum dots.^1^

These toughening approaches fundamentally involve molecular-level interactions through bonded interactions such as covalent bonds and nonbonded interactions: hydrogen bonds, ionic interactions, π–π interactions, and van der Waals interactions.^2−6^ For example, Cheng et al.^6^ developed a three component thermoset that improved the tensile strength by 101%. The three components included the primary epoxy resin (diglycidyl ether bisphenol A, DGEBA), a linear amine hardener (hexamethylenediamine, HDA) and a multifunctional hardener (5-amino-1H-benzotriazole, BTraz). The introduction of BTraz initiated hierarchical hydrogen bonds in the polymer network which helped in dissipating energy. The 40% BTraz material generated the best tensile properties (strength = 117.7 MPa and elongation = 14.9%). In a separate study, similar approach was implemented by inducing multiple supramolecular mechanisms.^5^ A 2-phenylimidazole (PID) molecule was added to a DGEBA resin to promote π-π stacking, hydrogen bonding and ionic bond formation.^5^ The results showed that a PID molar ratio of 0.6 in the epoxy generated a tensile strength of 65.4 MPa (a 54% increase) but reduced the strain at break. With a PID molar ratio of 0.5 the recorded toughness was the highest at 164.7 MJ/m^3^ compared to the unreinforced system at 17 MJ/m^3^.

In addition to tailored thermoset chemistries that enhance mechanical behavior, nanomaterials are used to strengthen and toughen polymers. These nanomaterials include clay, rubber, glass, and carbon-based materials.^7−10^ For example, clay fillers (at ∼5% wt) can increase tensile modulus and strength by ∼38 and ∼42%, respectively, in epoxy.^11−13^ In contrast, rubber (<10% wt) reduces epoxy stiffness of epoxy by 3–10% and strength by 2–20%;^14−17^ but it enhances the toughness by 300% with a rubber particle size of ∼15 nm.^18^

Carbon nanotubes (CNTs) and graphene nanoplatelets (GNPs) can enhance the tensile properties of epoxy at relatively low filler fractions (<5% wt).^19−30^ These enhancements range from 2–30% in stiffness and 10–60% in strength. While beneficial at optimal concentrations, these fillers can cause issues like poor bonding, agglomeration, voids, misalignment, and waviness in the nanocomposite.^8,31−33^ Achieving these improvements depends on consistent filler quality and preparation, filler–matrix chemical compatibility, composite fabrication processes, accurate measurements, and other human factors.

Moreover, hybrid composites with multiple fillers of different sizes (e.g., combination of CNTs and GNPs) have shown significant improvements in properties.^19,22−24,26−28,34−36^ However, they produce numerous fabrication challenges stemming from low dispersion due to filler agglomeration. One way to mitigate agglomeration is to tailor the surface of the nanomaterials with various functional groups. For instance, oxidized GNPs better disperse in epoxy matrices and show enhanced properties compared to pristine GNPs.^14,21,25,30,37^ Similar findings were reported with functionalized CNTs.^22,28,34,38−40^ These functional groups help enhance interaction with the matrix resulting in a stronger interface. One study reported enhanced stiffness and strength of polyether ether ketone reinforced with carbon fibers.^41^ The carbon fibers were coated with functionalized CNTs with carboxyl groups and poly(ether imide). The functionalized CNTs and poly(ether imide) introduced π–π stacking and hydrogen bonding with the matrix that enhances the properties without adversely impacting the matrix properties.^41^ Zhang et al.^42^ investigated the effects of including functionalized CNTs in an epoxy-carbon fiber composite. Two CNTs were used to survey the extent of property enhancement. It was reported that the CNTs with carboxylic acid groups (54 and 59% increase in modulus and strength) generated better results than aminated CNTs (36 and 36% increase in modulus and strength). Further observations revealed that carboxylic acid groups promoted better packing of polymer chains. One unique approach that enhances matrix stiffness and strength while enhancing toughness is to use quantum dots.

Quantum-dots (QDs) are nanofillers with sizes smaller than 20 nm, which is much smaller than any other nanofillers. Due to their size, QDs provide higher surface–surface contact with the matrix and enable more opportunities to promote molecular interactions like hydrogen bonds. In addition, quantum dots are photoluminescent, which makes the QD nanocomposites automatically multifunctional. For example, carbon-dot containing epoxy is shown to act as strain sensor.^43^ Moreover, graphene quantum dots (GQDs) are more effective in increasing thermal conductivity compared to GNPs.^44^ Also, GQDs can be used with other larger fillers to create hybrid multiscale composites without significantly changing the matrix viscosity.

QDs were reported to enhance the mechanical properties of thermosets.^37,44−53^ Gogoi et al.^47^ reported an increase in mechanical strength from 5.8 to 28.8 MPa by introducing 1.5 wt % of carbon QDs in a polyurethane matrix; also, a 30% increase in elongation at break and a 300% increase in toughness was observed. In another study, Karimi et al.^37^ used 0.1 wt % of ∼2 nm sized graphene oxide QDs (GOQDs) to enhance epoxy polymer properties. Compared to the graphene oxide (GO) filled resin, the increase in strength and stiffness was 69 and 27% respectively. The reported properties were even higher than those of the neat resin. Moreover, MoS2 QDs enhanced epoxy fracture toughness by 81%, flexural strength by 66%, stiffness by 6%;^46^ epoxy was composed of diglycidyl ether of bisphenol A (DGEBA) and diamino diphenylmethane (DDM). The simultaneous enhancement of strength, toughness, and stiffness are rare in material systems yet MoS2 QDs resulted in such behavior through nonbonded interactions below 1 wt % fraction.

Furthermore, Gobi et al.^45^ fabricated a GQD-epoxy nanocomposite and reported an increase of 125% in strength and increase of 153% in modulus for porous epoxy. The epoxy-GQD nanocomposite displayed reduced performance beyond 2.5 wt % GQD due to agglomeration of ∼16 nm diameter GQDs. Another study reported increased strength of epoxy by ∼25% using GQDs of ∼18 nm diameter; further field emission scanning electron microscopy characterization showed uniform distribution of the GQDs in the matrix.^51^ Note that, in this study an increase in cross-link density with the addition of 5 wt % GQDs was also observed, the surface chemistry of the GQDs were unknown.^51^ The reinforcing effect of graphene based QDs can be attributed to the high dispersibility in epoxy polymers which enable intermolecular interactions through large surface areas.

Despite the benefits, the underlying reasons for these enhanced mechanical properties with QDs are unknown. Specifically, the surface chemistry, diameter, number of layers, wt %, mixing techniques of the QDs affect the nanocomposite properties. At the nanoscale, molecular dynamics (MD) simulations arise as the main approach to understanding the molecular mechanisms behind improved properties. MD was chosen over other modeling techniques, such as Monte Carlo (MC) or finite element methods (FEM), due to its ability to capture atomic-level interactions and time-dependent processes. Unlike MC, which is suited for equilibrium property evaluations, or FEM, which are ideal for macroscopic continuum modeling, MD enables dynamic simulations at the nanoscale with reactive force fields to account for chemical bonding changes. This makes MD particularly suitable for investigating the mechanical behavior of functionalized graphene quantum dot nanocomposites.

MD simulations have been used to predict the mechanical properties of epoxies and effect of hydrogen bonding on the properties of polymers.^42,54−56^ He et al.^54^ designed a high-performance epoxy material with increased hydrogen bond capabilities. The novel material displayed a 52.5% increase in flexural modulus (5.1 GPa) compared to a commercial analog (3.4 GPa). This enhancement was principally attributed to the reduction in free volume due to close packing of the polymer chains. Another study affirming this conclusion was reported by Li et al.^55^ Three epoxy resins were modeled to investigate hydrogen-bond-based stiffening. The material with the lowest free volume displayed more hydrogen bond formation and the highest tensile modulus of 2.45 GPa (∼25% more than the other resins).

MD was also used to investigate the effect of covalent bonds in aminated-GQD epoxy nanocomposites.^57^ Keles et al.^57−59^ reported aminated-GQDs improve epoxy stiffness by 6% and strength by 17%. Computational studies provide a common trend of property enhancements by improved polymer packing and reduction in free volume pockets in the material. Nonbonded interactions like hydrogen bonds, electrostatic interactions, van der Waals forces, and π–π stacking are credited for reducing the free volume.

Still, the effects of oxidized GQDs on the epoxy nanocomposite structure and mechanical properties are unknown. To guide future experimental investigations, here for the first time, we performed MD simulations that uncovered the effects of both bonded and nonbonded interactions through epoxide, carboxylic acid, and hydroxyl functionalization of the GQDs in a bisphenol-F epoxy matrix. Various GQD configurations were modeled by manipulating the functional groups’ locations at the GQD edge and surface. Ten different GQD-epoxy nanocomposites were synthesized, characterized, and tensile tested using reactive MD, a total of 50 nanocomposites and 150 tensile tests.

## 2 Modeling Methodology

**Figure 1:**
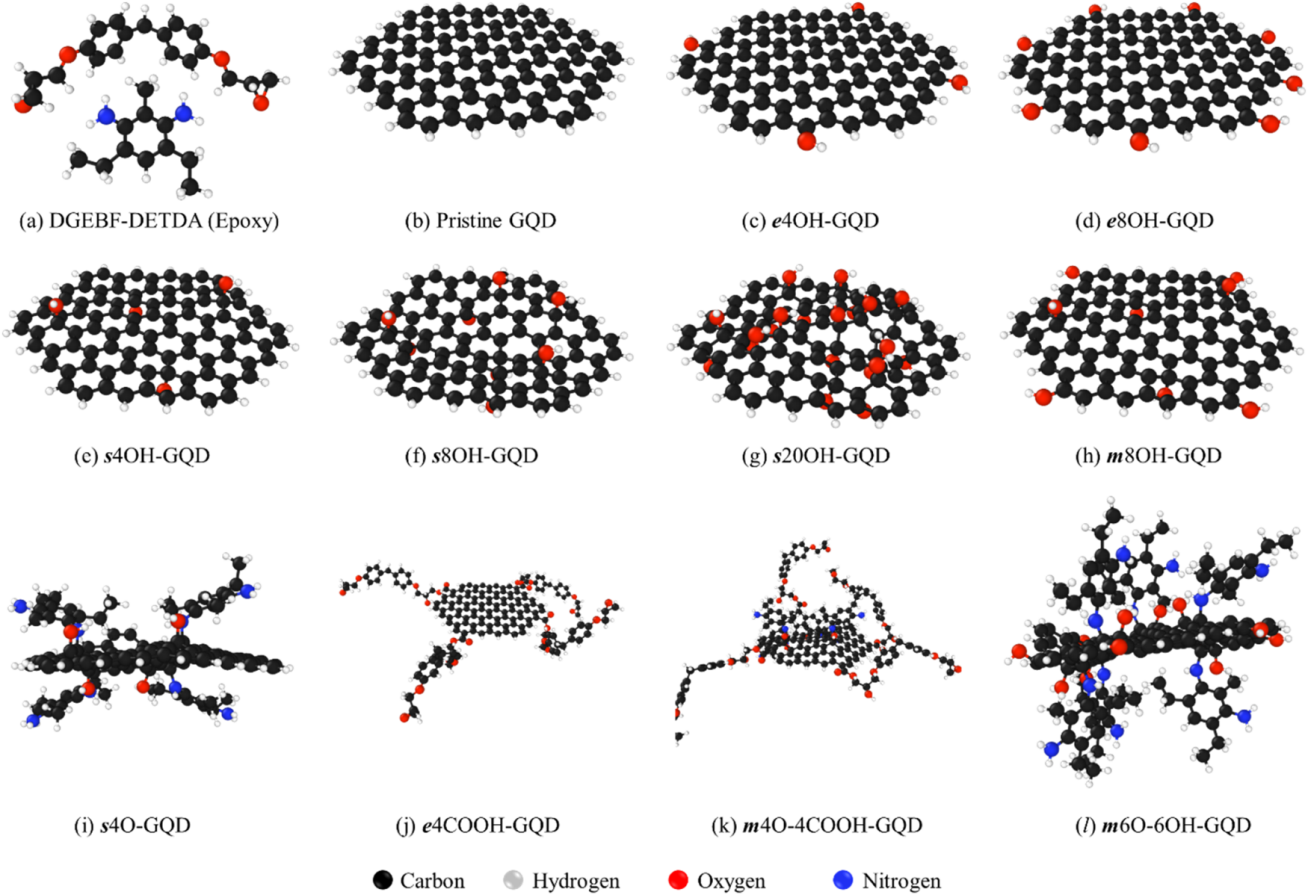
Individual chemistries of epoxy, pristine GQD, and oxidized GQDs.

The modeled polymer matrix was EPON 862 resin (diglycidyl ether bisphenol F or DGEBF) and Epikure W (diethyl toluene diamine or DETDA) as the curing agent. The material is a two-component thermoset with a difunctional resin monomer and tetra-functional hardener monomer. Figure 1a shows the molecular structure of the two components and Figure 1b–l shows the GQD structures. The GQD models were oxidized by attaching epoxide, carboxyl acid, and hydroxyl functional groups. A total of ten unique chemistries were explored. The properties of neat epoxy and pristine GQD-epoxy systems^57^ were used to benchmark the predictions. The ten modeled GQDs can be broadly classified into two groups – bonded and nonbonded GQDs. The nonbonded GQDs structures are shown in Figure 1b–h. The bonded GQD structures are shown in Figure 1i–l, which also depict the crosslinked polymer chains. The locations of the functional groups are GQD edge (***e***), GQD surface (***s***) and a mixture of edge and surface or (***m***). All the functional groups and their locations on the GQD were carefully chosen as reported in the literature.^37,60^Table 1 provides the atomic composition of the different GQD structures. For each GQD structure, five replicates were built with identical chemical compositions but varying distributions. The 12 material systems studied are as follows:

a. **Epoxy**: neat bisphenol-F diglycidyl ether (DGEBF) and diethyl toluene diamine (DETDA) as shown in Figure 1a.
b. **GQD**: hydrogen terminated GQD as shown in Figure 1b.
c. ***e*****4OH-GQD**: hydroxylated GQD with four hydroxyl groups on the edge and rest of the edge carbons terminated by hydrogen as shown in Figure 1c.
d. ***e*****8OH-GQD**: hydroxylated GQD with eight hydroxyl groups on the edge and rest of the edge carbons terminated by hydrogen as shown in Figure 1d.
e. ***s*****4OH-GQD**: hydroxylated GQD with four hydroxyl groups on the surface and the edge carbons terminated by hydrogen as shown in Figure 1e.
f. ***s*****8OH-GQD**: hydroxylated GQD with eight hydroxyl groups on the surface and the edge carbons terminated by hydrogen as shown in Figure 1f.
g. ***s*****20OH-GQD**: hydroxylated GQD with 20 hydroxyl groups on the surface and the edge carbons terminated by hydrogen as shown in Figure 1g.
h. ***m*****8OH-GQD**: hydroxylated GQD with four hydroxyl groups on the edge and on the surface; rest of the edge carbons terminated by hydrogen as shown in Figure 1h.
i. ***s*****4O-GQD**: epoxidized GQD with four epoxide rings on the surface and the edge carbons terminated by hydrogen as shown in Figure 1i.
j. ***e*****4COOH-GQD**: carboxylic acid GQD with four groups on the edge and the rest of the edge carbons terminated by hydrogen as shown in Figure 1j.
k. ***m*****4O-4COOH-GQD**: oxidized GQD with four epoxide rings on the surface, four carboxylic acid groups on the edges, and the rest of the edge carbons terminated by hydrogen as shown in Figure 1k.
l. ***m*****6O-6OH-GQD**: oxidized GQD with six epoxide rings on the surface, six hydroxyl groups on the edges, and rest of the edge carbons terminated by hydrogen as shown in Figure 1l.

**Table 1:** Atomic Composition of Oxidized GQDs–Carbon (C), Hydrogen (H), and Oxygen (O)

material	C(sp^2^) %	C(sp^3^) %	H(-R) %	H(−OH) %	O (epoxide) %	O(−OH) %	O(=O) %
pristine GQD	80	0	20	0	0	0	0
*e*4OH-GQD	77.4	0	16.1	3.2	0	3.2	0
*e*8OH-GQD	75	0	12.5	6.3	0	6.3	0
*s*4OH-GQD	71.9	3.1	18.8	3.1	0	3.1	0
*s*8OH-GQD	64.7	5.9	17.6	5.9	0	5.9	0
*s*20OH-GQD	47.5	12.5	15	12.5	0	12.5	0
*m*8OH-GQD	69.7	3	15.2	6.1	0	6.1	0
*s*4O-GQD	71	6.5	19.4	0	3.2	0	0
*e*4COOH-GQD	72.7	3	15.2	3	0	3	3
*m*4O-4COOH-GQD	64.7	8.8	14.7	2.9	2.9	2.9	2.9
*m*6O-6OH-GQD	63.6	9.1	13.6	4.5	4.6	4.6	0

The all-atom simulations were performed using LAMMPS open-source software.^61^ The Interface force field based on the polymer-consistent force field (PCFF-IFF) was used to model the interatomic interactions for all the individual molecular structures during initialization and cross-linking.^62,63^ For mechanical simulations, the ReaxFF force field was used to accurately capture the molecular response to the applied strain.^64^ All the MD simulations were performed on Anvil, a high performance computing cluster (HPC) at Purdue University, Indiana, USA^65^ through NSF ACCESS^66^ and on the College of Engineering HPC at San Jose State University, California, USA. The LUNAR package was used to construct the starting structures.^67^ LUNAR is an open-source, standalone toolkit which stands for LAMMPS Utility (for) Network Analysis (and) Reactivity. Using this toolkit, PCFF-IFF parameters where assigned to the molecules and cross-linking files for REACTER^68^ were generated. REACTER was used to simulate chemical cross-linking. The individual epoxy monomers were mixed in a simulation box with a mixing ratio of 2:1. This selected ratio ensures maximum cross-linking in the system.^69^ The cross-linking reaction for all the systems involved the two-step amine-epoxide reaction which is well documented.^70^ The GQD structure was inserted into a mixture of ∼5600 polymer atoms. The total number of models generated was 50 (10 unique GQDs with 5 replicates each).

To produce the bulk system including the GQD, the model was densified to the liquid density of the polymer. The system density was increased from 0.09 – 0.10 to 1.17 g/cm^3^.^69^ The simulation was performed using the NVT ensemble at room temperature conditions. A Nose-Hoover thermostat and a 1 fs (fs) time step was implemented with a constant deformation rate of 10 Å/ns. The dense model was annealed by cooling the system from 500 to 300 K with a constant cooling rate of 50 K/ns. Postannealing, the model was cross-linked by elevating the system temperature to 500 K. REACTER was implemented to achieve a fully cross-linked system. The average cross-link density for all the models is listed in Table 2. Out of the ten modeled GQD-epoxy systems, the *s*4O-GQD, *e*4COOH-GQD, *m*4O-4COOH-GQD, and *m*6O-6OH-GQD models were the only ones with covalent bonds present between the GQD and epoxy atoms. The cross-linked models were reannealed using the same settings as before. Next, the model was relaxed using the NPT ensemble at 300 K temperature and 1 atm pressure. The relaxed model was imported into ReaxFF by rerelaxing it at 300 K temperature and 1 atm pressure.

**Table 2:** MD Prediction of Physical and Mechanical Properties- Young’s Modulus (*E*), Yield Strength (σ_yt_), Poisson’s Ratio (ν), Fractional Free Volume (FFV), and Tensile Strain (ε)

					ν	FFV		
model	cross-link density (%)	mass density (g/cm^3^)	*E* (GPa)	σ_yt_ (MPa)	ν	ε = 0%	ε = 10%	change(%)
epoxy^57^	81.77 ± 1.55	1.226 ± 0.003	2.94 ± 0.06	109.4 ± 1.59	0.42 ± 0.01	1.76 ± 0.05	2.41 ± 0.08	37.39
GQD^57^	86.88 ± 0.83	1.226 ± 0.001	2.64 ± 0.05	103.42 ± 1.57	0.38 ± 0.01	1.95 ± 0.06	2.37 ± 0.05	21.24
*e*4OH-GQD	86.98 ± 1.08	1.239 ± 0.003	3.18 ± 0.04	117.73 ± 1.70	0.33 ± 0.01	1.83 ± 0.10	2.42 ± 0.09	32.07
*e*8OH-GQD	84.69 ± 0.73	1.237 ± 0.002	2.98 ± 0.06	118.25 ± 2.51	0.4 ± 0.01	1.87 ± 0.07	2.51 ± 0.10	34.55
*s*4OH-GQD	89.58 ± 1.23	1.233 ± 0.003	3.27 ± 0.05	121.37 ± 2.10	0.38 ± 0.01	2.01 ± 0.14	2.52 ± 0.08	25.88
*s*8OH-GQD	85.42 ± 0.00	1.233 ± 0.003	3.48 ± 0.05	132.69 ± 1.79	0.32 ± 0.01	1.77 ± 0.10	2.39 ± 0.03	34.68
*s*20OH-GQD	84.17 ± 0.71	1.236 ± 0.004	2.74 ± 0.03	170.76 ± 1.37	0.31 ± 0.01	1.86 ± 0.11	2.58 ± 0.09	38.23
*m*8OH-GQD	90.31 ± 0.97	1.232 ± 0.004	3.42 ± 0.07	134.72 ± 1.86	0.35 ± 0.01	2.13 ± 0.12	2.79 ± 0.10	30.78
*s*4O-GQD	91.8 ± 1.84	1.235 ± 0.002	3.19 ± 0.05	129.51 ± 2.77	0.35 ± 0.01	1.90 ± 0.12	2.64 ± 0.06	39.00
*e*4COOH-GQD	85.20 ± 1.30	1.233 ± 0.003	2.91 ± 0.04	145.47 ± 2.18	0.37 ± 0.01	2.09 ± 0.09	2.47 ± 0.07	18.22
*m*4O-4COOH-GQD	85.0 ± 0.83	1.229 ± 0.002	3.15 ± 0.05	145.26 ± 3.13	0.35 ± 0.01	2.16 ± 0.07	2.62 ± 0.05	21.11
*m*6O-6OH-GQD	84.90 ± 0.00	1.233 ± 0.003	3.1 ± 0.06	108.65 ± 1.34	0.35 ± 0.01	2.11 ± 0.20	2.42 ± 0.05	14.89

The fully equilibrated models were then simulated for the mechanical response. A uniaxial strain was applied to the models in the *x-*, *y-*, and *z-*directions. Thus, for each model, three stress–strain responses were computed. Therefore, for the 50 material systems, a total of 150 stress–strain data sets were generated. A strain rate of 2 × 10^8^ s^–1^ was applied with a 0.1 fs time step. The individual stress–strain data was generated by exporting the average virial stress data for the model.

Free volume analyses were performed using the LUNAR package. LUNAR uses the grid-probe approach to compute the free volume in an MD simulation box.^71^ The governing principle is identical to the positron annihilation lifetime spectroscopy (PALS) measurement, where positrons (diameter = 1–1.1 Å) are inserted into a material and are annihilated upon contact with electrons.^72^ If voids are present, the positrons have a longer lifetime, which is measured to establish the relative amount of free volume in the material. In MD, a probe or dummy atom is inserted into the MD unit cell in user-defined locations or at grid points. The calculation is sensitive to the grid spacing and the probe diameter.^73,74^ For a given set of these parameters, the free volume is defined by the locations in which the probe does not overlap with any atoms. The atom diameter is defined by the van der Waals radii provided by the force field. For this study, a grid spacing of 0.3 Å and a probe diameter of 0.6 Å were used. The selected probe size confirmed exclusion of empty pockets within the phenyl rings and similar intramolecular spaces from the free volume calculation.^75^

## 3 Results and Discussion

We simulated 50 different nanocomposites with 10 unique GQD chemistries. The mass density of the models was computed with ReaxFF. The density predictions do not show any significant dependence on the GQD system. Table 2 lists all the density values for the different modeled systems. The predicted mass density values for the neat epoxy using the same ReaxFF parameter set has been reported by Keles et al.^57^ The experimentally measured mass density for this material is in the range of 1.193–1.200 g/cm^3^.^70,76,77^ The overprediction of the mass density using ReaxFF is well documented elsewhere.^62,69^ For the *s*8OH-GQD and *m*6O-6OH-GQD systems the computed standard deviation in cross-link density was zero since all the replicates displayed an identical number of bond formations. Although the cross-link density number was the same, the participating reactive sites varied between the replicates. Because all the systems displayed a high amount of cross-linking, the largest molecular network always embraced the simulation box boundaries, which ensured effective load transmission through the molecular network.

The mechanical properties for all 12 systems (including epoxy and pristine GQD) are included in Table 2. The MD predicted Young’s modulus (*E*) for all the oxidized GQD systems was in the range 1.43–5.76 GPa. Experimentally measured mechanical properties for the different GQD systems discussed herein are not available in the literature because no studies have been performed on these systems with the same exact chemistry. However, the epoxy resin used in this study has been studied extensively.^70,76,78^ The experimental value of *E* reported is in the range of 1.6–3.3 GPa.^70,76,78^ These measurements were recorded at experimental strain rates of 10^–4^–10^2^ s^–1^. Although the MD simulations employed a strain rate of 10^8^ s^–1^, the predicted stiffness is consistent with the previously published MD studies.^62,69,79,80^

Compared to neat epoxy, a maximum increase in *E* of 18.4% was computed from the GQD-epoxy models. The maximum predicted value was from the *s*8OH-GQD system (18.4%) with the *m*8OH-GQD system (16.3%) also displaying similar predictions. Both these systems involve a GQD-epoxy interface with hydroxyl groups across the basal plane. Some of the material systems with covalent bonds between the GQD and the epoxy also displayed a heightened stiffness. The *s*4O-GQD system showed an increase in stiffness (8.5%) and the *e*4COOH-GQD showed a decrease in stiffness (1.0%). The addition of four carboxylic acid groups in the *m*4O-4COOH-GQD system did not provide any improvement over the *s*4O-GQD. The *m*6O-6OH-GQD system also showed an improvement in stiffness (5.4%).

According to one study, the binding energy of a polymer molecule and flat sp^2^ surface is higher than that in the transverse direction of the surface.^81^ Binding energy is defined as the energy difference between the case where two molecules are adjacent and the case where they are not. Higher binding energy means higher interaction. ReaxFF simulations revealed higher binding energy of DETDA molecule and a flattened CNT at the CNT surface over the rounded edge.^81^ Since the sp^2^ graphitic surface is energetically more favorable than the edge, the GQD-polymer interactions were stronger at the surface. Furthermore, the addition of hydroxyl groups on the surface amplifies the interfacial interaction by forming hydrogen bonds.^82,83^ Computational studies between pristine graphene and graphene oxide suggest epoxy monomers prefer hydroxyl groups.^82,83^ Similarly, the addition of hydroxyl groups on the GQD surface (*s*4OH-GQD, *s*8OH-GQD, s20OH-GQD, and *m*8OH-GQD) enabled intermolecular interactions between the GQDs and the epoxy atoms. In all the models, hydrogen bonds were formed between the hydroxyl groups on the GQDs and the hydroxyl (from reacted epoxide) and amine groups in the epoxy. A decrease in stiffness (10.2%) was also observed in the pristine GQD models.^57^ But also, with the *s*20OH-GQD models a decrease in stiffness (6.8%) was observed. This decrement can be a result of oversaturation of the GQD surface which was not observed with the other surface hydroxylated GQDs.

The MD predicted yield strength (σ_yt_) for all the systems was in the range 27.98–272.70 MPa. The experimental value for the neat epoxy is in the range 36–90 MPa.^70,76,78^ Unlike the stiffness results, the maximum strength increases of 56.1% were displayed by the *s*20OH-GQD material system compared to neat epoxy. In comparison, the *m*8OH-GQD model showed a 23.1% increase and the *s*8OH-GQD model showed a 21.3% increase. The rest of the systems showed relatively lower enhancements. The pristine GQD model was the only system which showed a reduction in the strength of the epoxy by 5.5% and the *m*6O-6OH-GQD showed a ∼ 0% change. Overall, the nonbonded systems with hydroxyl groups on the basal plane (*s*4OH-GQD, *s*8OH-GQD, *s*20OH-GQD, and *m*8OH-GQD) displayed higher strength output. Again, the presence of functional groups at energetically favorable locations aided in the increased strength predictions.^81^ For the bonded models, the increases in strength were promising from the GQD models with the carboxylic acid groups on the edges. Both the *e*4COOH-GQD and *m*4O-4COOH-GQD systems showed an increase of 32.8 and 33.0% respectively. The Poisson’s ratio (ν) remained unaffected by the different configurations of GQD and ranged between 0.31–0.42.

Comparing the three hydroxylated GQD cases – edge, surface and mixed functionalization, the surface models showed highest increases in the mechanical properties. The *e*8OH-GQD models showed the lowest enhancements, lower than *e*4OH-GQD models. The higher amount of functionalization on the edges was detrimental to the mechanical response of the epoxy. On the contrary, with the *m*8OH-GQD the hydroxyl groups were present on the edges and the surface and the addition of the four groups on the surface bumped up the stiffness and strength by 7.5 and 14.4% compared to the *e*4OH-GQD models. The surface for all the hydroxylated GQDs predominantly consists of sp^2^ carbons which in addition to hydrogen bonds also enable π-π stacking with phenyl rings in the polymer and attract electronegative groups like amines. The *s*20OH-GQD models included an oversaturated surface with 20 hydroxyl groups. The enhancements were significant in terms of strength but none in stiffness. The *s*20OH-GQDs made the material elastic but tough.

The *s*4O-GQD and *m*6O-6OH-GQD systems include epoxide groups on the surface that form a covalent bond with the epoxy. The presence of covalent bonds mildly enhances the mechanical properties of the epoxy. The nonbonded systems with hydroxyl groups display better mechanical properties. The *m*6O-6OH-GQD model predicted no improvement in strength and small increase in stiffness. This material system consisted of the highest number of functional groups on surface and edges. The oversaturation of the functional groups caused steric hindrances which impacted the mechanical properties. Like the *s*4O-GQD, the *m*4O-4COOH-GQD also consists of four surface epoxide groups but additionally also includes four carboxylic acid groups on the edges like *e*4COOH-GQD. The inclusion of the edge carboxylic acid groups does have a positive impact on the yield strength of the nanocomposite.

**Figure 2:**
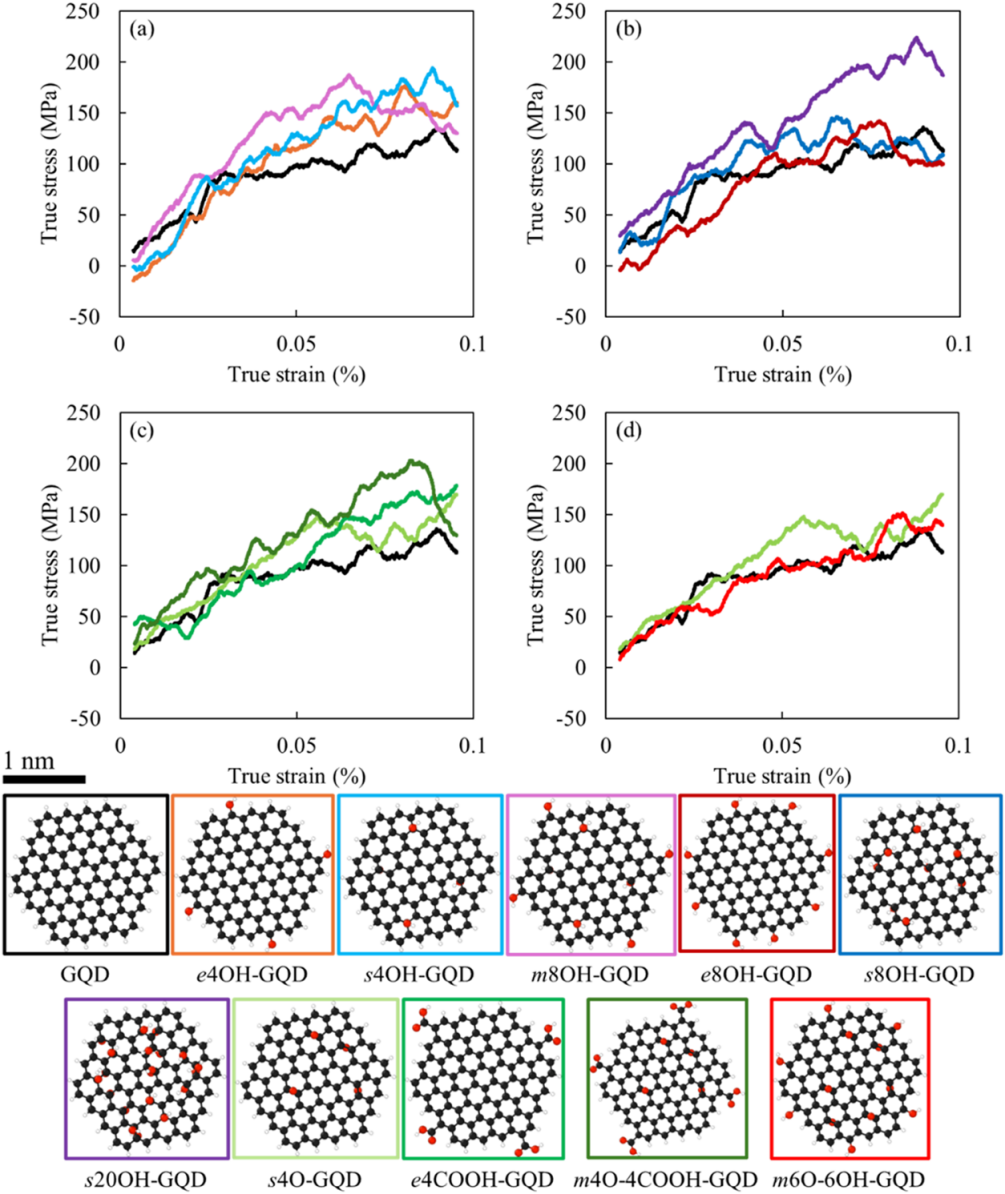
(a)–(d) Representative stress–strain response for all the GQD systems, with the color coding showing the GQD nanostructure for each stress–strain response in the bottom. (For interpreting the color references in this figure, the reader is referred to the web version of this article.)

Integration of a pristine GQD with the epoxy resulted in a decrease of stiffness and strength. The pristine GQD provided minimal intermolecular interaction due to lack of any chemical groups that provide bonded and nonbonded exchanges. The GQD functioned more as a defect than enhancement.^57^Figure 2 shows the stress–strain response of the representative GQD-epoxy systems along with the individual molecular models of oxidized GQD and the corresponding color coded borders. Representative stress–strain response curves in Figure 2 were selected based on average responses across replicates to ensure reliability and reproducibility. Figure 2a shows the comparison of mechanical response from *e*4OH-GQD, *s*4OH-GQD, *m*8OH-GQD, and the pristine GQD systems. The mechanical response from the *m*8OH-GQD is substantially better than the other three GQD models. This difference can be attributed to the greater number of functional groups and the location of the functional groups on the basal plane of the GQD. Figure 2b compares the *e*8OH-GQD, *s*8OH-GQD, *s*20OH-GQD, and the pristine GQD systems. The *e*8OH-GQD results show some stiffer regions in the lower strain zone, but the strength remains relatively unaffected by the inclusion of the functional groups on the GQD edge. The same cannot be said about the *s*8OH-GQD and *s*20OH-GQD models with surface groups. The increase in the yield strength is extensive, albeit the stiffness for the *s*20OH-GQD models remains unaffected.

Figure 2c includes the stress–strain response from the covalently bonded GQD models *s*4O-GQD, *e*4COOH-GQD, and *m*4O-4COOH-GQD with the pristine GQD system. The *s*4O-GQD results show some improvement in mechanical properties over the pristine GQD case, but with the *e*4COOH-GQD system the increase in strength is more profound. The *m*4O-4COOH-GQD models combine the two configurations and show greater improvement in both *E* and σ_yt_. Figure 2d compares the pristine GQD with the *s*4O-GQD, and *m*6O-6OH-GQD systems. The *m*6O-6OH-GQD system shows no effect of functionalization on the mechanical response of the nanocomposite. The *m*6O-6OH-GQD models involved the highest branching at the GQD surface due to the covalent bonding between the epoxide groups and the hardener molecules. Postreaction, the attached hardener can still react with the epoxy molecule to complete the two-step epoxide-amine reaction, thus increasing the atom density in the interfacial region. The covalent bonds at the interface restrict optimal molecular conformations and create steric hindrances. In comparison, although the *s*20OH-GQD model consists of the highest functional groups, the nonbonded configuration allows unrestricted atomic movements and enables molecular interactions like hydrogen bonding. In general, oxidizing the GQD by different oxygen groups (epoxide, carboxylic acid, and hydroxyl) helps enhance the interface between the epoxy and GQD. The mechanical property predictions show that the hydroxyl groups on the surface and the carboxylic acid groups on the edges provide good material enhancements.

Keles et al.^57^ observed that the orientations of graphene quantum dots (GQDs) did not have a significant impact on the mechanical properties of the nanocomposites. This is likely due to the effective dispersion and uniform intermolecular interactions promoted by the GQDs within the epoxy matrix, which mitigate the effects of anisotropy. For the present work, similar analyses were performed to verify this observation and confirmed that the output properties exhibit minimal dependence on GQD orientation.

The free volume was computed for all the simulated models at 0% strain and at 10% strain. Free volume was computed to confirm polymer chain packing due to heightened intermolecular interaction and the effective mechanical response.^54,55^Table 2 lists the average fractional free volume (FFV) for all the GQD systems along with the change in the FFV due to the 10% strain. The FFV for the equilibrated models was in the range of 1.50–2.77%. An experimental study on a similar epoxy system was conducted by Jean et al.^84^ For bisphenol A epoxy with an aromatic amine curing agent (DGEBA-DDS), a FFV of 1.51–1.82% was reported which validates the predicted FFV of neat epoxy in this study (1.62–1.90%). Also, Li et al.^75^ used a similar grid-probe approach to compute the FFV for the exact same epoxy (DGEBF-DETDA) MD model. An FFV of 2–4% was reported in slightly different relaxation settings.^75^

**Figure 3:**
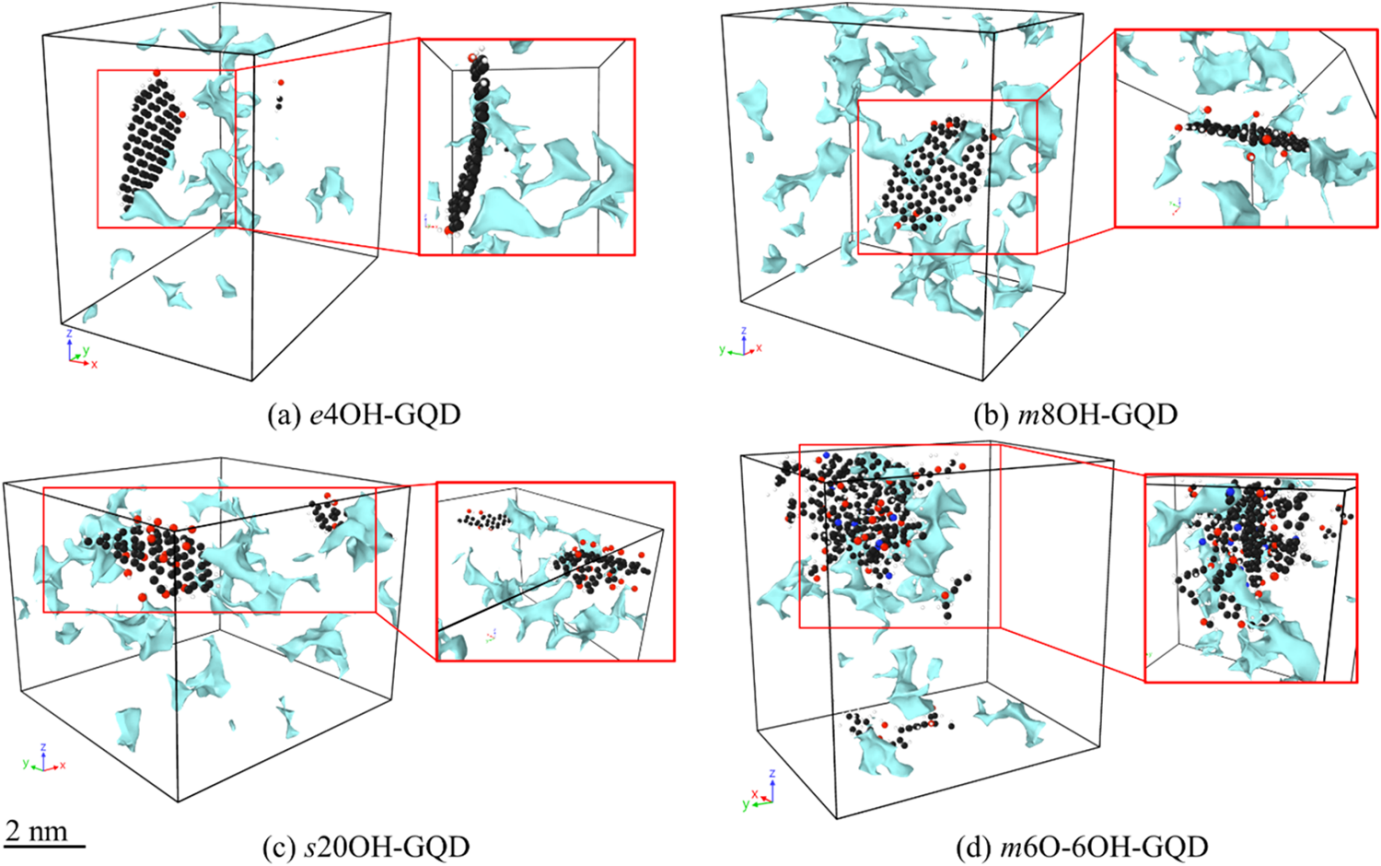
Location of large free volume voids in the MD simulation cell with the GQD location for (a) e4OH-GQD,(b) m8OH-GQD, (c) s20OH-GQD, and (d) m6O-6OH-GQD.

The nonbonded GQD systems which include the pristine GQD and edge, surface, and mixed hydroxylated GQDs displayed lower FFV than the covalent bonded GQDs. The edge hydroxylated GQDs displayed lower FFV than the surface and mixed models. Since the functional groups were concentrated on the edges, the pristine sp^2^ carbon surface of the GQD allowed for better packing of polymer chains. The neat packing enabled lower free volume pockets. With the surface functional groups, the flat GQD surface was disturbed by the poking hydroxyl groups. The higher density surface models helped reduce the free volume by pulling more polymer atoms toward the surface. Overall, the mixed *m*8OH-GQD predicted the highest FFV due to the large span of the GQD. The presence of hydroxyl groups on edge and surface resulted in greater geometric variability and hence more avenues for void generation. The covalently bonded GQD systems recorded higher FFV values when compared to the nonbonded GQD systems. The rigid networks created poor atomic conformations near the interface. Moreover, the interfacial bond density has a negative influence on the FFV in the system. The lowest density of interfacial bonds was in the *s*4O-GQD models which showed lower FFV. The denser systems like the *m*4O-4COOH-GQD and *m*6O-6OH-GQD display the highest FFV. Figure 3 shows the location of large voids in the MD simulation cell for select models. From Figure 3a–c it is evident that the voids were developed in locations where hydroxyl groups were absent. For example, with edge models the voids around the GQD were along the surface and vice-a-versa can be said for the surface models. In case Figure 3d, the covalent bonds on the GQD surface resulted in undesirable atomic conformations which generated pockets all around the GQD.

**Figure 4:**
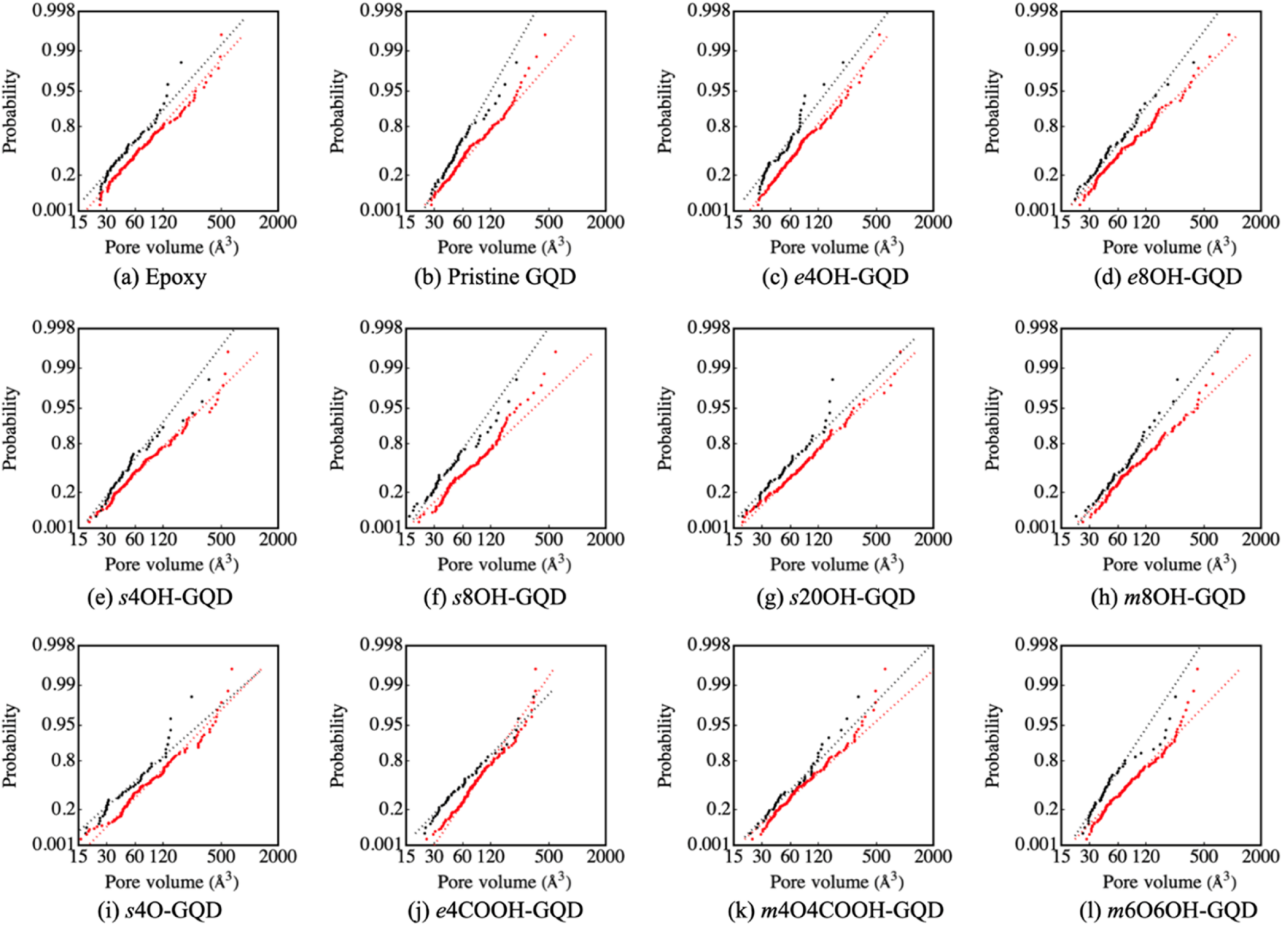
Probability plots using Fréchet distribution of ten largest voids in all the modeled systems. Void distribution at 0% strain in black and at 10% strain in red. (For interpreting the color references in this figure, the reader is referred to the web version of this article.)

**Figure 5:**
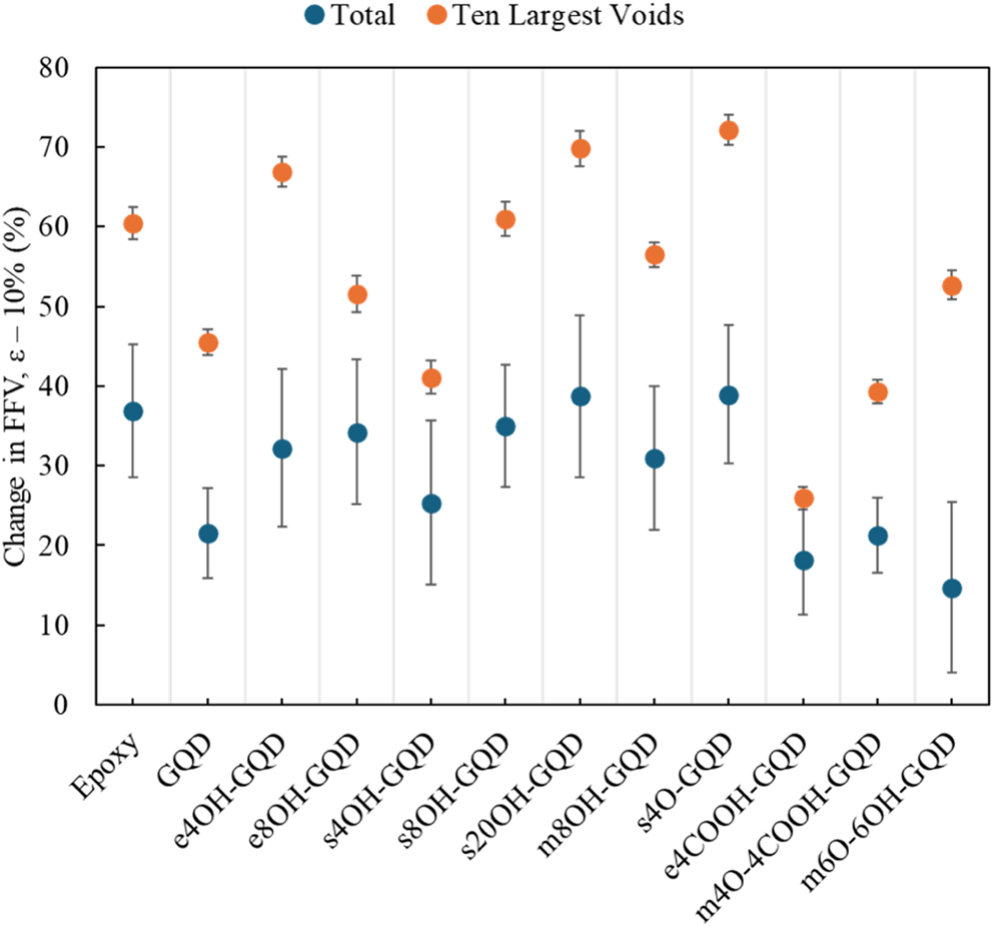
Deformation-induced change in FFV. (For interpreting the color references in this figure, the reader is referred to the web version of this article.)

Figure 4 shows the probability plots of the ten highest free volume voids for all the models, 0% strain in red and 10% strain in black. The GQDs with carboxylic acid groups display no change in void volume and corresponding sizes due to tension. The largest effect of tension on the FFV was observed in the *s*4O-GQD and *s*20OH-GQD systems. Post tension, the *m*6O-6OH-GQD models showed the lowest amount of FFV change (14.89%). However, comparing the change in FFV for the ten largest voids (Figure 5) it was observed that the FFV increases by 50% which is comparable to the rest of the hydroxylated GQD systems. This indicated that the GQD model with the higher covalent bond based branching generated larger voids when put under tension. For the other systems the applied strain to the system resulted in different sized voids, among which the carboxyl acid group GQDs produced the lowest changes in void volume.

The effect of deformation on the FFV content was more apparent in the nonbonded models as seen in Figure 5. The unconfined nature of the hydroxylated GQDs resulted in more void generation when the model was deformed. The most increase was observed in the *s*20OH-GQD system. The hydroxyl groups present in higher density induced advanced molecular instability at the interface. The growth in larger voids was about 70%. In the *s*4O-GQD and *m*6O-6OH-GQD systems, the covalent networks were concentrated on the surface producing stiffer topologies. The large voids in these systems grew by more than 50%. The same growth was less than 40% for the carboxylic acid or edge functionalized GQD systems. The covalent networks on the GQD edges pushed the polymer atoms in outward direction from the GQD center.

Further simulation and experimental investigations are needed to reveal the effect of quantum dot surface chemistry on the mechanical behavior of thermosets and the underlying molecular mechanisms for strengthening, stiffening, and toughening. A recent study showed that covalent bonding is effective in enhancing mechanical properties of a two-dimensional (2D) covalent organic framework made through the bonding of 2,5-dihydroxyterethaldehyde (DHTA) and 1,3,5-tris(4-aminophenyl)benzene (TAPB), which had a fracture strength of ∼ 750 MPa and stiffness of 10 GPa.^85^ This shows that the large chemical space of quantum dots and thermosets need further investigations to reveal the best performing nanocomposites. A machine learning based framework can be used to scan this chemical space to accelerate explorations toward next-generation applications.

## 4 Conclusions

In this study, we simulated 50 nanocomposites with various GQD chemistries and found that functionalized GQDs significantly improved the mechanical properties of epoxy-based composites. Surface hydroxylated GQDs provided the highest increases in stiffness and yield strength due to enhanced intermolecular interactions, while oversaturation of functional groups could reduce stiffness. The findings underscore the importance of functional group type and placement in optimizing composite properties. The following conclusions can be drawn from the current MD study:

1. Surface hydroxylated GQDs increase both Young’s modulus and yield strength. The s20OH-GQD showed a peak increase in yield strength of 56.1% but reduced stiffness by 6.8%. Conversely, s8OH-GQD exhibited both increased strength (21.3%) and stiffness (18.4%).
2. Edge hydroxylated GQDs did not significantly affect mechanical properties. The mixed hydroxylated GQD (with both edge and surface hydroxyl groups) showed an increase in Young’s modulus (16.3%) and yield strength (23.1%), indicating that surface functionalization plays a more crucial role.
3. Hydroxylated GQDs generally exhibited the lowest free volume. The mixed hydroxylated GQD had the highest free volume due to its extensive span and induced conflicting polymer chain alignments, creating voids.
4. Covalent bonding generally improved mechanical properties except for the m6O-6OH-GQD system. Surface-bonded GQDs showed limited improvement due to polymer chain attachment limiting contact. The m4O-4COOH-GQD model showed the best improvement (7.1% increase in stiffness and 33.0% increase in strength).
5. Nonbonded GQDs with hydroxyl groups on the basal plane outperformed covalently bonded GQDs in terms of mechanical property enhancement. The appropriate amount of surface functionalization can lead to more stable nanostructures.

Intelligent design of covalent networks in multi-GQD models is essential to maximize mechanical properties. Nonbonded GQDs with optimal surface functionalization are viable candidates for fillers, introducing supramolecular interactions akin to multicomponent polymer resins with multifunctional additives while enhancing stiffness through strong graphene lattice. Further studies are needed to discover the QD chemistries that improve the mechanical properties of the recyclable vitrimers, thermoplastics, and other multiscale multifunctional composites.^86,87^

## References

[ref1] Mi X.; Liang N.; Xu H.; Wu J.; Jiang Y.; Nie B.; Zhang D. Toughness and its mechanisms in epoxy resins. Prog. Mater. Sci. 2022, 130, 100977 10.1016/j.pmatsci.2022.100977.

[ref2] Montarnal D.; Tournilhac F.; Hidalgo M.; Leibler L. Epoxy-based networks combining chemical and supramolecular hydrogen-bonding crosslinks. J. Polym. Sci., Part A:Polym. Chem. 2010, 48 (5), 1133–1141. 10.1002/pola.23870.

[ref3] Seo J.; Yui N.; Seo J.-H. Development of a supramolecular accelerator simultaneously to increase the cross-linking density and ductility of an epoxy resin. Chem. Eng. J. 2019, 356, 303–311. 10.1016/j.cej.2018.09.020.

[ref4] Sun P.; Li Y.; Qin B.; Xu J.-F.; Zhang X. Super Strong and Multi-Reusable Supramolecular Epoxy Hot Melt Adhesives. ACS Mater. Lett. 2021, 3 (7), 1003–1009. 10.1021/acsmaterialslett.1c00277.

[ref5] Wang C.; Zhang S.; Li S.; Zhang L.; Zhou Y.; Ma J.; Zhang L. Toughening rigid thermoset films via molecular enforced integration of covalent crosslinking and multiple supramolecular interactions. J. Polym. Sci. 2021, 59 (13), 1446–1455. 10.1002/pol.20210134.

[ref6] Wang C.; Zhang S.; Zhang L.; Xu Y.; Zhang L. Evading the strength–ductility trade-off dilemma of rigid thermosets by incorporating triple cross-links of varying strengths. Polym. Chem. 2020, 11 (39), 6281–6287. 10.1039/D0PY00928H.

[ref7] Rallini M.; Kenny J. M. Nanofillers in Polymers. In Modification of Polymer Properties; Elsevier, 2017; pp 47–86.

[ref8] Mousavi S. R.; Estaji S.; Paydayesh A.; Arjmand M.; Jafari S. H.; Nouranian S.; Khonakdar H. A. A review of recent progress in improving the fracture toughness of epoxy-based composites using carbonaceous nanofillers. Polym. Compos. 2022, 43 (4), 1871–1886. 10.1002/pc.26518.

[ref9] Balguri P. K.; Samuel D. G. H.; Thumu U. A review on mechanical properties of epoxy nanocomposites. Mater. Today: Proc. 2021, 44, 346–355. 10.1016/j.matpr.2020.09.742.

[ref10] Kumar S. M.; Kamal S. Effect of Carbon Nanofillers on the Mechanical and Interfacial Properties of Epoxy Based Nanocomposites: A Review. Polym. Sci., Ser. A 2019, 61 (4), 439–460. 10.1134/S0965545X19040096.

[ref11] Wang K.; Chen L.; Wu J.; Toh M. L.; He C.; Yee A. F. Epoxy Nanocomposites with Highly Exfoliated Clay: Mechanical Properties and Fracture Mechanisms. Macromolecules 2005, 38 (3), 788–800. 10.1021/ma048465n.

[ref12] Pappas J.; Patel K.; Nauman E. B. Structure and properties of phenolic resin/nanoclay composites synthesized by in situ polymerization. J. Appl. Polym. Sci. 2005, 95 (5), 1169–1174. 10.1002/app.21303.

[ref13] Wang M.; Fan X.; Thitsartarn W.; He C. Rheological and mechanical properties of epoxy/clay nanocomposites with enhanced tensile and fracture toughnesses. Polymer 2015, 58, 43–52. 10.1016/j.polymer.2014.12.042.

[ref14] Gong L.-X.; Zhao L.; Tang L.-C.; Liu H.-Y.; Mai Y.-W. Balanced electrical, thermal and mechanical properties of epoxy composites filled with chemically reduced graphene oxide and rubber nanoparticles. Compos. Sci. Technol. 2015, 121, 104–114. 10.1016/j.compscitech.2015.10.023.

[ref15] Tang L.-C.; Wang X.; Wan Y.-J.; Wu L.-B.; Jiang J.-X.; Lai G.-Q. Mechanical properties and fracture behaviors of epoxy composites with multi-scale rubber particles. Mater. Chem. Phys. 2013, 141 (1), 333–342. 10.1016/j.matchemphys.2013.05.018.

[ref16] Bajpai A.; Wetzel B.; Klingler A.; Friedrich K. Mechanical properties and fracture behavior of high-performance epoxy nanocomposites modified with block polymer and core–shell rubber particles. J. Appl. Polym. Sci. 2020, 137 (11), 48471 10.1002/app.48471.

[ref17] Quan D.; Ivankovic A. Effect of core–shell rubber (CSR) nano-particles on mechanical properties and fracture toughness of an epoxy polymer. Polymer 2015, 66, 16–28. 10.1016/j.polymer.2015.04.002.

[ref18] Liu J. D.; Sue H.-J.; Thompson Z. J.; Bates F. S.; Dettloff M.; Jacob G.; Verghese N.; Pham H. Effect of crosslink density on fracture behavior of model epoxies containing block copolymer nanoparticles. Polymer 2009, 50 (19), 4683–4689. 10.1016/j.polymer.2009.05.006.

[ref19] Ayatollahi M. R.; Shadlou S.; Shokrieh M. M. Fracture toughness of epoxy/multi-walled carbon nanotube nano-composites under bending and shear loading conditions. Mater. Des. 2011, 32 (4), 2115–2124. 10.1016/j.matdes.2010.11.034.

[ref20] Hsieh T. H.; Kinloch A. J.; Taylor A. C.; Kinloch I. A. The effect of carbon nanotubes on the fracture toughness and fatigue performance of a thermosetting epoxy polymer. J. Mater. Sci. 2011, 46 (23), 7525–7535. 10.1007/s10853-011-5724-0.

[ref21] Tang L.-C.; Wan Y.-J.; Yan D.; Pei Y.-B.; Zhao L.; Li Y.-B.; Wu L.-B.; Jiang J.-X.; Lai G.-Q. The effect of graphene dispersion on the mechanical properties of graphene/epoxy composites. Carbon 2013, 60, 16–27. 10.1016/j.carbon.2013.03.050.

[ref22] Tang L.-c.; Zhang H.; Han J.-h.; Wu X.-p.; Zhang Z. Fracture mechanisms of epoxy filled with ozone functionalized multi-wall carbon nanotubes. Compos. Sci. Technol. 2011, 72 (1), 7–13. 10.1016/j.compscitech.2011.07.016.

[ref23] Zhou Y. X.; Wu P. X.; Cheng Z. Y.; Ingram J.; Jeelani S. Improvement in electrical, thermal and mechanical properties of epoxy by filling carbon nanotube. Express Polym. Lett. 2008, 2 (1), 40–48. 10.3144/expresspolymlett.2008.6.

[ref24] Rafiee M. A.; Rafiee J.; Wang Z.; Song H.; Yu Z.-Z.; Koratkar N. Enhanced Mechanical Properties of Nanocomposites at Low Graphene Content. ACS Nano 2009, 3 (12), 3884–3890. 10.1021/nn9010472. 19957928

[ref25] Wan Y.-J.; Gong L.-X.; Tang L.-C.; Wu L.-B.; Jiang J.-X. Mechanical properties of epoxy composites filled with silane-functionalized graphene oxide. Composites, Part A 2014, 64, 79–89. 10.1016/j.compositesa.2014.04.023.

[ref26] Ahmadi-Moghadam B.; Sharafimasooleh M.; Shadlou S.; Taheri F. Effect of functionalization of graphene nanoplatelets on the mechanical response of graphene/epoxy composites. Mater. Des. 2015, 66, 142–149. 10.1016/j.matdes.2014.10.047.

[ref27] Gojny F.; Wichmann M.; Fiedler B.; Schulte K. Influence of different carbon nanotubes on the mechanical properties of epoxy matrix composites – A comparative study. Compos. Sci. Technol. 2005, 65 (15–16), 2300–2313. 10.1016/j.compscitech.2005.04.021.

[ref28] Sun L.; Warren G. L.; O’Reilly J. Y.; Everett W. N.; Lee S. M.; Davis D.; Lagoudas D.; Sue H. J. Mechanical properties of surface-functionalized SWCNT/epoxy composites. Carbon 2008, 46 (2), 320–328. 10.1016/j.carbon.2007.11.051.

[ref29] Qi B.; Lu S. R.; Xiao X. E.; Pan L. L.; Tan F. Z.; Yu J. H. Enhanced thermal and mechanical properties of epoxy composites by mixing thermotropic liquid crystalline epoxy grafted graphene oxide. Express Polym. Lett. 2014, 8 (7), 467–479. 10.3144/expresspolymlett.2014.51.

[ref30] Baruah P.; Karak N. Bio-based tough hyperbranched epoxy/graphene oxide nanocomposite with enhanced biodegradability attribute. Polym. Degrad. Stab. 2016, 129, 26–33. 10.1016/j.polymdegradstab.2016.03.021.

[ref31] Varischetti J.; Jang J.-S.; Gibson R. F.; Suhr J. Effect of filler waviness and orientation on the damping behavior of CNF-reinforced epoxy composites. J. Mater. Sci. 2013, 48 (2), 832–840. 10.1007/s10853-012-6803-6.

[ref32] Chatterjee S.; Nafezarefi F.; Tai N. H.; Schlagenhauf L.; Nüesch F. A.; Chu B. T. T. Size and synergy effects of nanofiller hybrids including graphene nanoplatelets and carbon nanotubes in mechanical properties of epoxy composites. Carbon 2012, 50 (15), 5380–5386. 10.1016/j.carbon.2012.07.021.

[ref33] Prolongo S. G.; Moriche R.; Jiménez-Suárez A.; Sánchez M.; Ureña A. Advantages and disadvantages of the addition of graphene nanoplatelets to epoxy resins. Eur. Polym. J. 2014, 61, 206–214. 10.1016/j.eurpolymj.2014.09.022.

[ref34] Zhu Y.; Bakis C. E.; Adair J. H. Effects of carbon nanofiller functionalization and distribution on interlaminar fracture toughness of multi-scale reinforced polymer composites. Carbon 2012, 50 (3), 1316–1331. 10.1016/j.carbon.2011.11.001.

[ref35] Hernández-Pérez A.; Avilés F.; May-Pat A.; Valadez-González A.; Herrera-Franco P. J.; Bartolo-Pérez P. Effective properties of multiwalled carbon nanotube/epoxy composites using two different tubes. Compos. Sci. Technol. 2008, 68 (6), 1422–1431. 10.1016/j.compscitech.2007.11.001.

[ref36] Gkikas G.; Barkoula N. M.; Paipetis A. S. Effect of dispersion conditions on the thermo-mechanical and toughness properties of multi walled carbon nanotubes-reinforced epoxy. Composites, Part B 2012, 43 (6), 2697–2705. 10.1016/j.compositesb.2012.01.070.

[ref37] Karimi B.; Ramezanzadeh B. A comparative study on the effects of ultrathin luminescent graphene oxide quantum dot (GOQD) and graphene oxide (GO) nanosheets on the interfacial interactions and mechanical properties of an epoxy composite. J. Colloid Interface Sci. 2017, 493, 62–76. 10.1016/j.jcis.2017.01.013. 28088122

[ref38] Cheng Q.; Wang B.; Zhang C.; Liang Z. Functionalized carbon-nanotube sheet/bismaleimide nanocomposites: mechanical and electrical performance beyond carbon-fiber composites. Small 2010, 6 (6), 763–767. 10.1002/smll.200901957. 20183814

[ref39] Wang S.; Liang Z.; Liu T.; Wang B.; Zhang C. Effective amino-functionalization of carbon nanotubes for reinforcing epoxy polymer composites. Nanotechnology 2006, 17 (6), 1551–1557. 10.1088/0957-4484/17/6/003. 26558557

[ref40] Gojny F. H.; Wichmann M. H. G.; Köpke U.; Fiedler B.; Schulte K. Carbon nanotube-reinforced epoxy-composites: enhanced stiffness and fracture toughness at low nanotube content. Compos. Sci. Technol. 2004, 64 (15), 2363–2371. 10.1016/j.compscitech.2004.04.002.

[ref41] Hassan E. A. M.; Yang L.; Elagib T. H. H.; Ge D.; Lv X.; Zhou J.; Yu M.; Zhu S. Synergistic effect of hydrogen bonding and π-π stacking in interface of CF/PEEK composites. Composites, Part B 2019, 171, 70–77. 10.1016/j.compositesb.2019.04.015.

[ref42] Zhang W.; Deng X.; Sui G.; Yang X. Improving interfacial and mechanical properties of carbon nanotube-sized carbon fiber/epoxy composites. Carbon 2019, 145, 629–639. 10.1016/j.carbon.2019.01.063.

[ref43] Sherburne M. D.; Roberts C. R.; Brewer J. S.; Weber T. E.; Laurvick T. V.; Chandrahalim H. Comprehensive Optical Strain Sensing Through the Use of Colloidal Quantum Dots. ACS Appl. Mater. Interfaces 2020, 12 (39), 44156–44162. 10.1021/acsami.0c12110. 32877159

[ref44] Seibert J. R.; Keleş Ö.; Wang J.; Erogbogbo F. Infusion of graphene quantum dots to modulate thermal conductivity and dynamic mechanical properties of polymers. Polymer 2019, 185, 121988 10.1016/j.polymer.2019.121988. PMC6940706 31909111

[ref45] Gobi N.; Vijayakumar D.; Keles O.; Erogbogbo F. Infusion of Graphene Quantum Dots to Create Stronger, Tougher, and Brighter Polymer Composites. ACS Omega 2017, 2 (8), 4356–4362. 10.1021/acsomega.6b00517. 31457728 PMC6641722

[ref46] Riaz S.; Park S.-J. A comparative study on nanoinclusion effect of MoS2 nanosheets and MoS2 quantum dots on fracture toughness and interfacial properties of epoxy composites. Composites, Part A 2021, 146, 106419 10.1016/j.compositesa.2021.106419.

[ref47] Gogoi S.; Kumar M.; Mandal B. B.; Karak N. High performance luminescent thermosetting waterborne hyperbranched polyurethane/carbon quantum dot nanocomposite with in vitro cytocompatibility. Compos. Sci. Technol. 2015, 118, 39–46. 10.1016/j.compscitech.2015.08.010.

[ref48] De B.; Voit B.; Karak N. Carbon dot reduced Cu2O nanohybrid/hyperbranched epoxy nanocomposite: mechanical, thermal and photocatalytic activity. RSC Adv. 2014, 4 (102), 58453–58459. 10.1039/C4RA11120F.

[ref49] Nejad S. S.; Babaie A.; Bagheri M.; Rezaei M.; Abbasi F.; Shomali A. Effects of graphene quantum dot (GQD) on photoluminescence, mechanical, thermal and shape memory properties of thermoplastic polyurethane nanocomposites. Polym. Adv. Technol. 2020, 31 (10), 2279–2289. 10.1002/pat.4948.

[ref50] Bagheri M.; Mahmoodzadeh A. Polycaprolactone/Graphene Nanocomposites: Synthesis, Characterization and Mechanical Properties of Electrospun Nanofibers. J. Inorg. Organometall. Polym. Mater. 2020, 30 (5), 1566–1577. 10.1007/s10904-019-01340-8.

[ref51] Madhi A.; Hadavand B. S. Fluorescent epoxy-graphene quantum dots nanocomposites: synthesis and study of properties. Polym.-Plast. Technol. Mater. 2022, 61 (2), 117–130. 10.1080/25740881.2021.1959929.

[ref52] Zhang C.; Du L.; Liu C.; Li Y.; Yang Z.; Cao Y.-C. Photostable epoxy polymerized carbon quantum dots luminescent thin films and the performance study. Results Phys. 2016, 6, 767–771. 10.1016/j.rinp.2016.10.013.

[ref53] Bai J.; Ren W.; Wang Y.; Li X.; Zhang C.; Li Z.; Xie Z. High-performance thermoplastic polyurethane elastomer/carbon dots bulk nanocomposites with strong luminescence. High Perform. Polym. 2020, 32 (7), 857–867. 10.1177/0954008320907123.

[ref54] He J.; Li L.; Zhou J.; Tian J.; Chen Y.; Zou H.; Liang M. Ultra-high modulus epoxy resin reinforced by intensive hydrogen bond network: From design, synthesis, mechanism to applications. Compos. Sci. Technol. 2023, 231, 109815 10.1016/j.compscitech.2022.109815.

[ref55] Li W.; Ma J.; Wu S.; Zhang J.; Cheng J. The effect of hydrogen bond on the thermal and mechanical properties of furan epoxy resins: Molecular dynamics simulation study. Polym. Test. 2021, 101, 107275 10.1016/j.polymertesting.2021.107275.

[ref56] Sheng C.; Wu G.; Sun X.; Liu S. Molecular Dynamics Investigation of the Thermo-Mechanical Properties of the Moisture Invaded and Cross-Linked Epoxy System. Polymers 2022, 14 (1), 103 10.3390/polym14010103. PMC8747217 35012124

[ref57] Keleş Ö.; Deshpande P. P. Mechanical behavior of graphene quantum dot epoxy nanocomposites: A molecular dynamics study. Mater. Lett. 2024, 362, 136206 10.1016/j.matlet.2024.136206. PMC10881529 38389955

[ref58] Deshpande P. P.; Keles O. Simulation data for engineering graphene quantum dot epoxy nanocomposites using molecular dynamics. Data Brief 2024, 53, 110169 10.1016/j.dib.2024.110169. 38389955 PMC10881529

[ref59] Deshpande P. P.; Keleş Ö. In Effect of Graphene Quantum Dots on the Mechanical Properties of Bisphenol F-Based Epoxy, American Society for Composites 2023, 2023.

[ref60] Bokare A.; Nordlund D.; Melendrez C.; Robinson R.; Keles O.; Wolcott A.; Erogbogbo F. Surface functionality and formation mechanisms of carbon and graphene quantum dots. Diamond Relat. Mater. 2020, 110, 108101 10.1016/j.diamond.2020.108101.

[ref61] Thompson A. P.; Aktulga H. M.; Berger R.; Bolintineanu D. S.; Brown W. M.; Crozier P. S.; Veld P. J. i.; Kohlmeyer A.; Moore S. G.; Nguyen T. D.; Shan R.; Stevens M. J.; Tranchida J.; Trott C.; Plimpton S. J. LAMMPS - a flexible simulation tool for particle-based materials modeling at the atomic, meso, and continuum scales. Comput. Phys. Commun. 2022, 271, 108171 10.1016/j.cpc.2021.108171.

[ref62] Odegard G. M.; Patil S. U.; Deshpande P. P.; Kanhaiya K.; Winetrout J. J.; Heinz H.; Shah S. P.; Maiaru M. Molecular Dynamics Modeling of Epoxy Resins Using the Reactive Interface Force Field. Macromolecules 2021, 54 (21), 9815–9824. 10.1021/acs.macromol.1c01813.

[ref63] Winetrout J. J.; Kanhaiya K.; Sachdeva G.; Pandey R.; Damirchi B.; van Duin A.; Odegard G.; Heinz H. Implementing Reactivity in Molecular Dynamics Simulations with the Interface Force Field (IFF-R) and Other Harmonic Force Fields. 2021. 10.1038/s41467-024-50793-0 PMC11391066 39261455

[ref64] Zhang W.; van Duin A. C. T. Improvement of the ReaxFF Description for Functionalized Hydrocarbon/Water Weak Interactions in the Condensed Phase. J. Phys. Chem. B 2018, 122 (14), 4083–4092. 10.1021/acs.jpcb.8b01127. 29518340

[ref65] Song X. C.; Smith P.; Kalyanam R.; Zhu X.; Adams E.; Colby K.; Finnegan P.; Gough E.; Hillery E.; Irvine R.; Maji A.; John J. S. Anvil - System Architecture and Experiences from Deployment and Early User Operations. In Practice and Experience in Advanced Research Computing.; Association for Computing Machinery: Boston, MA, USA, 2022.

[ref66] Boerner T. J.; Deems S.; Furlani T. R.; Knuth S. L.; Towns J. ACCESS: Advancing Innovation: NSF’s Advanced Cyberinfrastructure Coordination Ecosystem: Services & Support. In Practice and Experience in Advanced Research Computing 2023: Computing for the Common Good; Association for Computing Machinery: Portland, OR, USA, 2023; pp 173–176.

[ref67] Kemppainen J.; Gissinger J. R.; Gowtham S.; Odegard G. M. LUNAR: Automated Input Generation and Analysis for Reactive LAMMPS Simulations. J. Chem. Inf. Model. 2024, 64, 5108–5126. 10.1021/acs.jcim.4c00730. 38926930 PMC11234336

[ref68] Gissinger J. R.; Jensen B. D.; Wise K. E. REACTER: A Heuristic Method for Reactive Molecular Dynamics. Macromolecules 2020, 53 (22), 9953–9961. 10.1021/acs.macromol.0c02012.

[ref69] Radue M. S.; Jensen B. D.; Gowtham S.; Klimek-McDonald D. R.; King J. A.; Odegard G. M. Comparing the Mechanical Response of Di-, Tri-, and Tetra-functional Resin Epoxies with Reactive Molecular Dynamics. J. Polym. Sci., Part B:Polym. Phys. 2018, 56 (3), 255–264. 10.1002/polb.24539. PMC6894187 31806922

[ref70] Patil S. U.; Shah S. P.; Olaya M.; Deshpande P. P.; Maiaru M.; Odegard G. M. Reactive Molecular Dynamics Simulation of Epoxy for the Full Cross-Linking Process. ACS Appl. Polym. Mater. 2021, 3 (11), 5788–5797. 10.1021/acsapm.1c01024.

[ref71] Kemppainen J.; Gissinger J.; Gowtham S.; Odegard G. LUNAR: Automated Input Generation and Analysis for Reactive LAMMPS Simulations ChemRxiv 2024 10.26434/chemrxiv-2024-9tqz2. PMC11234336 38926930

[ref72] Jean Y. C. Positron annihilation spectroscopy for chemical analysis: A novel probe for microstructural analysis of polymers. Microchem. J. 1990, 42 (1), 72–102. 10.1016/0026-265X(90)90027-3.

[ref73] Hofmann D.; Heuchel M.; Yampolskii Y.; Khotimskii V.; Shantarovich V. Free Volume Distributions in Ultrahigh and Lower Free Volume Polymers: Comparison between Molecular Modeling and Positron Lifetime Studies. Macromolecules 2002, 35 (6), 2129–2140. 10.1021/ma011360p.

[ref74] Jansen J. C.; Macchione M.; Tocci E.; De Lorenzo L.; Yampolskii Y. P.; Sanfirova O.; Shantarovich V. P.; Heuchel M.; Hofmann D.; Drioli E. Comparative Study of Different Probing Techniques for the Analysis of the Free Volume Distribution in Amorphous Glassy Perfluoropolymers. Macromolecules 2009, 42 (19), 7589–7604. 10.1021/ma901244d.

[ref75] Li C.; Strachan A. Free volume evolution in the process of epoxy curing and its effect on mechanical properties. Polymer 2016, 97, 456–464. 10.1016/j.polymer.2016.05.059.

[ref76] Littell J. D.; Ruggeri C. R.; Goldberg R. K.; Roberts G. D.; Arnold W. A.; Binienda W. K. Measurement of Epoxy Resin Tension, Compression, and Shear Stress–Strain Curves over a Wide Range of Strain Rates Using Small Test Specimens. J. Aerosp. Eng. 2008, 21 (3), 162–173. 10.1061/(ASCE)0893-1321(2008)21:3(162).

[ref77] Klimek-McDonald D. R.; King J. A.; Miskioglu I.; Pineda E. J.; Odegard G. M. Determination and Modeling of Mechanical Properties for Graphene Nanoplatelet/Epoxy Composites. Polym. Compos. 2018, 39 (6), 1845–1851. 10.1002/pc.24137.

[ref78] Gilat A.; Goldberg R. K.; Roberts G. D. Strain Rate Sensitivity of Epoxy Resin in Tensile and Shear Loading. J. Aerosp. Eng. 2007, 20 (2), 75–89. 10.1061/(ASCE)0893-1321(2007)20:2(75).

[ref79] Vashisth A.; Ashraf C.; Zhang W.; Bakis C. E.; van Duin A. C. T. Accelerated ReaxFF Simulations for Describing the Reactive Cross-Linking of Polymers. J. Phys. Chem. A 2018, 122 (32), 6633–6642. 10.1021/acs.jpca.8b03826. 29996044

[ref80] Li C.; Strachan A. Molecular simulations of crosslinking process of thermosetting polymers. Polymer 2010, 51 (25), 6058–6070. 10.1016/j.polymer.2010.10.033.

[ref81] Damirchi B.; Radue M.; Kanhaiya K.; Heinz H.; Odegard G. M.; van Duin A. C. T. ReaxFF Reactive Force Field Study of Polymerization of a Polymer Matrix in a Carbon Nanotube-Composite System. J. Phys. Chem. C 2020, 124 (37), 20488–20497. 10.1021/acs.jpcc.0c03509.

[ref82] Pathak A. K.; Dhakate S. R. Validation of experimental results for graphene oxide-epoxy polymer nanocomposite through computational analysis. J. Polym. Sci. 2021, 59 (1), 84–99. 10.1002/pol.20200442.

[ref83] Shrestha A.; Sumiya Y.; Okazawa K.; Uwabe T.; Yoshizawa K. Molecular Understanding of Adhesion of Epoxy Resin to Graphene and Graphene Oxide Surfaces in Terms of Orbital Interactions. Langmuir 2023, 39 (15), 5514–5526. 10.1021/acs.langmuir.3c00262. 37027214

[ref84] Jean Y. C.; Deng Q.; Nguyen T. T. Free-Volume Hole Properties in Thermosetting Plastics Probed by Positron Annihilation Spectroscopy: Chain Extension Chemistry. Macromolecules 1995, 28 (26), 8840–8844. 10.1021/ma00130a018.

[ref85] Fang Q.; Sui C.; Wang C.; Zhai T.; Zhang J.; Liang J.; Guo H.; Sandoz-Rosado E.; Lou J. Strong and flaw-insensitive two-dimensional covalent organic frameworks. Matter 2021, 4 (3), 1017–1028. 10.1016/j.matt.2021.01.001.

[ref86] Yang Y.; Xu Y.; Ji Y.; Wei Y. Functional epoxy vitrimers and composites. Prog. Mater. Sci. 2021, 120, 100710 10.1016/j.pmatsci.2020.100710.

[ref87] Mittal G.; Rhee K. Y.; Mišković-Stanković V.; Hui D. Reinforcements in multi-scale polymer composites: Processing, properties, and applications. Composites, Part B 2018, 138, 122–139. 10.1016/j.compositesb.2017.11.028.

